# Case Report: Left Ventricular Unloading Using a Mechanical CPR Device in a Prolonged Accidental Hypothermic Cardiac Arrest Treated by VA-ECMO – a Novel Approach

**DOI:** 10.3389/fcvm.2021.707663

**Published:** 2021-06-24

**Authors:** Simon A. Amacher, Jonas Quitt, Eva Hammel, Urs Zenklusen, Ayham Darwisch, Martin Siegemund

**Affiliations:** ^1^Intensive Care Medicine, University Hospital Basel, Basel, Switzerland; ^2^Department of Cardiac Surgery, University Hospital Basel, Basel, Switzerland; ^3^Department of Clinical Research, University of Basel, Basel, Switzerland

**Keywords:** extracorporeal life support, accidental hypothermia, cardiac arrest, left ventricular unloading, harlequin syndrome, cardiopulmonary resuscitation

## Abstract

We recently treated a 36-year-old previously healthy male with a prolonged hypothermic (lowest temperature 22.3°C) cardiac arrest after an alcohol intoxication with a return of spontaneous circulation after 230min of mechanical cardiopulmonary resuscitation and rewarming by veno-arterial ECMO with femoral cannulation and retrograde perfusion of the aortic arch. Despite functional veno-arterial ECMO, we continued mechanical cardiopulmonary resuscitation (Auto Pulse™ device, ZOLL Medical Corporation, Chelmsford, USA) until return of spontaneous circulation to prevent left ventricular distention from persistent ventricular fibrillation. The case was further complicated by extensive trauma caused by mechanical cardiopulmonary resuscitation (multiple rib fractures, significant hemothorax, and a liver laceration requiring massive transfusion), lung failure necessitating a secondary switch to veno-venous ECMO, and acute kidney injury with the need for renal replacement therapy. Shortly after return of spontaneous circulation, the patient was already following commands and could be discharged 3 weeks later without neurologic, cardiac, or renal sequelae and being entirely well. Prolonged accidental hypothermic cardiac arrest might present with excellent outcomes when supported with veno-arterial ECMO. Until return of spontaneous circulation, one might consider continuing with mechanical cardiopulmonary resuscitation in addition to ECMO to allow some left ventricular unloading. However, the clinician should keep in mind that prolonged mechanical cardiopulmonary resuscitation may cause severe injuries.

## Introduction

Accidental hypothermic cardiac arrest (AHCA) usually results from exposure to freezing environments in high/low latitude regions, mountainous areas or drowning in icy water (1, 2). Progressive hypothermia leads to several physiological changes, such as reducing cardiac contractility and increased susceptibility to arrhythmias, finally resulting in cardiac arrest (3). Current guidelines recommend using extracorporeal life support (ECLS) to rewarm and resuscitate patients with accidental hypothermic cardiac arrest (4). AHCA treated by ECLS (veno-arterial Extracorporeal membrane oxygenation – VA-ECMO or conventional cardiopulmonary bypass - CPB) is associated with better survival and a favorable neurological outcome when compared with conventional cardiopulmonary resuscitation measures (2, 5). ECMO has several theoretical advantages over CPB: The possibility of prolonged extracorporeal support over days or even weeks (6), the feasibility of percutaneous cannulation under ongoing cardiopulmonary resuscitation (CPR) (7), systemic anticoagulation can be discontinued for several days, and the ECMO configuration can be switched to pulmonary support (veno-venous ECMO) only (8). However, one major disadvantage of VA-ECMO is the potentially detrimental effect on the left ventricle (LV) by progressive dilation. Hence, several LV unloading strategies have been described, consisting of a surgical or percutaneous LV vent (9–12). Herein, we present a novel approach of left ventricular unloading using a mechanical chest compression device in a case of a prolonged AHCA treated by veno-arterial extracorporeal membrane oxygenation (VA-ECMO) with an excellent outcome in a moderate climate (Basel, Switzerland).

## Case Description

**Figure 1:**
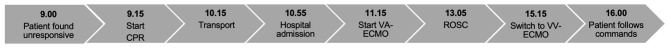
Resuscitation timeline. CPR, cardiopulmonary resuscitation; ROSC, Return of spontaneous circulation; ECMO, Extracorporeal membrane oxygenation; min, minutes; VA, veno-arterial; VV, veno-venous.

A 36-year-old previously healthy male of African descent was found by his neighbor in his house's backyard in south Germany at the border to Switzerland on the morning of an early winter day (12th of December). The lowest reported local temperature was −2°Celsius (°C) during nighttime. The patient was still moving but unresponsive and bradypnoeic. Emergency medical services arrived 15 min later upon the scene, detecting pulseless electrical activity and immediately implementing CPR. The initial measured body temperature at the scene was 23°C (tympanic thermometer). After prolonged CPR on-site, the patient was intubated and transported to our tertiary teaching hospital with a mechanical chest compression device (Auto Pulse™ device, ZOLL Medical Corporation, Chelmsford, USA) by ambulance. On admission, the patient was still in cardiac arrest showing asystole. The measured temperature on admission was 22.3°C. Transthoracic echocardiography (TTE) revealed cardiac standstill and no pericardial effusion. A venous blood gas analysis showed a severe metabolic acidosis with a pH of 6.81, lactate of 22 mmol/L, and a potassium of 3.2 mmol/L. Further, lab tests revealed a blood alcohol level of 2.3%0. Table 1 shows sequential blood gas analysis during resuscitation, and Figure 1 gives an overview of the resuscitation timeline.

**Table 1:** Timeline of sequential blood gas analysis.

**Parameters**	**Admission**	**After ECMO**	**Rewarming**	**1 min *before* ROSC**	**19 min *after* ROSC**	**VV-ECMO**	**VV-ECMO**
	**venous**	**arterial**	**arterial**	**arterial**	**arterial**	**arterial**	**arterial**
Time	**11:03**	**11:49**	**12:29**	**13:04**	13:34^*^	15:38^*^	**20:19**
Temperature (°Celsius)	22.3	26	30.1	34.6	35.9	35.8	35.7
PH	6.81	6.91	6.89	6.93	6.95	7.22	7.44
Bicarb (mmol/L)	10.7	6.1	5.3	5.2	9	17.1	20.7
BE	−24.6	−25.4	−26.4	−25.5	−21.5	−10	−2.3
PO2 (kpa)	9.4	29.7	22.7	18.6	8.6	10.9	15
PCO2 (kpa)	8.9	4	3.7	3.28	5.4	5.6	4.1
O2 Sat. (%)	68	98	97	96	72^**^	93	96
Lactate (mmol/L)	22	Not available	24	23	21	19	12.7
Potassium (mmol/L)	3.2	4.4	4.6	5.6	5.8	6.2	5
Glucose (mmol/L)	6.1	3.8	2.7	9.2	6.6	4	5
Hemoglobin (g/L)	151	108	103	82	74	89	107

*All arterial samples have been taken from the right radial artery. ROSC, Return of spontaneous circulation; ECMO, Extracorporeal membrane oxygenation min minutes; VV, veno-venous; kpa, kilopascal; mmol/L, millimoles per liter; g/L, grams per liter; PO2, partial pressure of oxygen; PCO2, partial pressure of carbon dioxide.*

* *Transfusion of four units of packed red blood cells and three units of fresh frozen plasma between 13:34 and 15:38.*

** *Low arterial oxygen saturation despite adequate pO2 due to rightwards shift of the oxygen binding curve*.

**Figure 2:**
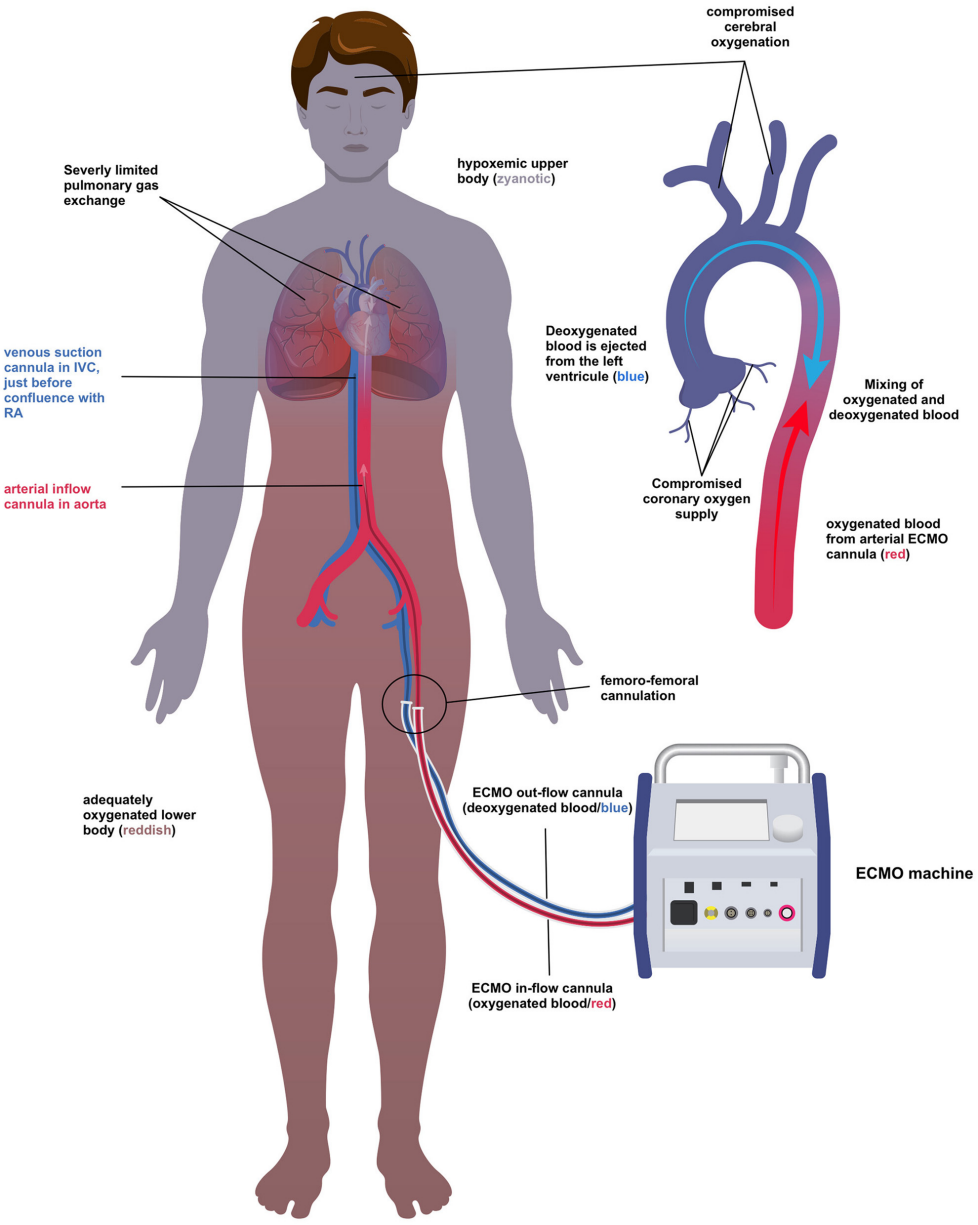
Differential hypoxemia. ECMO, Extracorporeal membrane oxygenation; IVC, inferior vena cava; RA, right atrium.

The patient was put on VA-ECMO with femoro-femoral cannulation under ongoing mechanical CPR in the emergency department. On ICU admission, the patient had already been resuscitated for approximately 2 h and now showed persistent ventricular fibrillation regardless of several defibrillation attempts after reaching 30°C body temperature. Despite a functional VA-ECMO, we continued mechanical CPR (in default setting; 80 compressions/min) intending to unload the left ventricle. After rewarming the patient's core temperature to 35°C, we successfully defibrillated the patient and achieved a return of spontaneous circulation (ROSC) after a total of 230 min of continuous CPR. Surprisingly, TTE revealed a well-coping heart with a recovering biventricular function shortly after ROSC. However, the patient developed differential hypoxemia (syn. Harlequin syndrome – Figure 2) due to the lungs' severely compromised oxygenation capacity jeopardizing cerebral and coronary oxygen supply. Given a well-coping heart, we placed an additional cannula in the right internal jugular vein and switched the ECMO uneventfully from veno-arterial to veno-venous configuration. After stabilization, a computed tomography scan unveiled severe bilateral atelectasis and a large unilateral hemothorax explaining the disturbed pulmonary gas exchange. Additionally, multiple bilateral rib fractures and a liver laceration with intra-abdominal free fluid were diagnosed. The hemothorax was treated by a chest drain, whereas the other injuries could be managed conservatively.

Three hours after ROSC, the patient was already following commands. VV - ECMO could be weaned successfully 2 days later. The further course was complicated by acute kidney injury requiring continuous veno-venous hemofiltration, ventilator-associated pneumonia and delirium. Seven days after admission, the patient could be extubated, showing no signs of neurological deficits. After extubation, TTE showed good biventricular cardiac function without signs of regional abnormalities. Eleven days after cardiac arrest, the patient was transferred to a regional hospital ward, where he was discharged a few days later, showing complete renal, cardiac, and neurologic recovery.

## Discussion

To our knowledge, this is the first description of left ventricular unloading achieved by continuous mechanical CPR with an excellent cardiac and neurologic outcome (6, 10–13). VA-ECMO support for refractory cardiac arrest can lead to progressive LV distension, especially in the context of aortic regurgitation: The retrograde perfusion of the aortic arch leads to progressive LV filling and left atrial inflow through Thebesian, and bronchial veins add to LV pressures and volume (12, 14–16). LV distension may result in pulmonary edema, prolonged cardiac arrest, and thromboembolic complications through blood stasis (12, 17–20). Finally, the additional cardiac injury limits the probability for complete myocardial recovery (21). Hence, it is essential to unload the LV during VA-ECMO in refractory hypothermic cardiac arrest until ROSC and echocardiographic evidence of aortic valve opening.

We decided to use a mechanical CPR device instead of a conventional surgical or percutaneous method to unload the LV in the presented case. This decision was based on the prolonged cardiac arrest caused by therapy refractory VF leading to a possibly futile situation and the logistic problem of intrahospital transport of an ECMO patient. After reaching 35°, defibrillation was finally successful with a rapidly recovering biventricular function, emphasizing the importance of LV unloading and adequate body temperature for regaining an output generating heart rhythm. In our case, this unconventional approach led to an excellent outcome, without any evidence of cardiac or neurologic sequelae. However, one must keep in mind that prolonged use of a mechanical chest compression device may have severe traumatic side-effects as in the presented case.

After ROSC, our patient presented with a classic Harlequin syndrome (Figure 2) (22). Cerebral and coronary perfusion in femoro-femoral VA-ECMO depends on the flow of oxygenated blood in the ascending aorta. In the presence of compromised pulmonary gas exchange, recovery of cardiac output opposite to the ECMO flow may lead to a critically reduced oxygen supply of the brain and heart. In such a situation, the options depend on the LV function. This case of hypothermic arrest had a normal biventricular function after ROSC, so we switched to VV- ECMO.

Ethanol intoxication during wintertime may have possible dramatic consequences, even in a moderate climate and a suburban area. Ethanol jeopardizes peripheral vasoconstriction, thereby, increasing the risk of severe hypothermia (23). AHCA patients are substantially different from conventional cardiac arrest patients concerning neurologic outcome due to the well-documented neuroprotective effects of profound hypothermia. Moreover, this case shows sequelae-free survival is possible even in a moderate climate (2, 5, 13, 24). To estimate survival probability after AHCA treated by ECLS, clinicians traditionally relied on potassium levels, as previous research showed an association between hyperkalemia and poor outcomes (25, 26). Nowadays, physicians can use the recently established HOPE score (Hypothermia Outcome Prediction after Extracorporeal Life Support for Hypothermic Cardiac Arrest Patients) for easier prognostication and resource allocation (27). The score was externally validated in 2019 and includes the following parameters (age, gender, presence of asphyxia, duration of CPR, temperature, and potassium on hospital admission) (28). Retrospectively, the HOPE score would have prognosticated a survival rate of 80% in our patient. Hence, we advocate using the HOPE score for prognostication in AHCA patients, as it can be easily calculated by a web tool: https://www.hypothermiascore.org/.

## Conclusion

In conclusion, we present an unconventional approach to LV venting in prolonged cardiac arrest treated by VA-ECMO with an excellent outcome. Due to the only anecdotal evidence of a single case report, the authors cannot recommend using mechanical CPR devices for LV unloading. Still, caregivers might consider it as an additional option in the field of ECMO-treated cardiac arrest.

## Reporting Guidelines

The case report follows the consensus-based clinical case reporting guideline (CARE) checklist (29).

## Data Availability Statement

The raw data supporting the conclusions of this article will be made available by the authors, without undue reservation.

## Ethics Statement

Written informed consent was obtained from the individual(s) for the publication of any potentially identifiable images or data included in this article.

## Author Contributions

JQ obtained informed consent from the patient. SA and JQ collected the presented clinical data and were responsible for the manuscript's writing and review process. AD and EH participated in the manuscript's review process. MS supervised the conceptualization, writing, and review process of the article. All authors have been involved in the patient's treatment.

## Conflict of Interest

The authors declare that the research was conducted in the absence of any commercial or financial relationships that could be construed as a potential conflict of interest.
